# Exploring Demographic, Physical, and Historical Explanations for the Genetic Structure of Two Lineages of Greater Antillean Bats

**DOI:** 10.1371/journal.pone.0017704

**Published:** 2011-03-21

**Authors:** Robert A. Muscarella, Kevin L. Murray, Derek Ortt, Amy L. Russell, Theodore H. Fleming

**Affiliations:** 1 Department of Ecology, Evolution and Environmental Biology, Columbia University, New York, New York, United States of America; 2 Department of Biology, University of Miami, Coral Gables, Florida, United States of America; 3 Rosenstiel School of Marine and Atmospheric Science, University of Miami, Miami, Florida, United States of America; 4 ImpactWeather, Houston, Texas, United States of America; 5 Department of Biology, Grand Valley State University, Allendale, Michigan, United States of America; University of Western Ontario, Canada

## Abstract

Observed patterns of genetic structure result from the interactions of demographic, physical, and historical influences on gene flow. The particular strength of various factors in governing gene flow, however, may differ between species in biologically relevant ways. We investigated the role of demographic factors (population size and sex-biased dispersal) and physical features (geographic distance, island size and climatological winds) on patterns of genetic structure and gene flow for two lineages of Greater Antillean bats. We used microsatellite genetic data to estimate demographic characteristics, infer population genetic structure, and estimate gene flow among island populations of *Erophylla sezekorni/E. bombifrons* and *Macrotus waterhousii* (Chiroptera: Phyllostomidae). Using a landscape genetics approach, we asked if geographic distance, island size, or climatological winds mediate historical gene flow in this system. Samples from 13 islands spanning *Erophylla*'s range clustered into five genetically distinct populations. Samples of *M. waterhousii* from eight islands represented eight genetically distinct populations. While we found evidence that a majority of historical gene flow between genetic populations was asymmetric for both lineages, we were not able to entirely rule out incomplete lineage sorting in generating this pattern. We found no evidence of contemporary gene flow except between two genetic populations of *Erophylla*. Both lineages exhibited significant isolation by geographic distance. Patterns of genetic structure and gene flow, however, were not explained by differences in relative effective population sizes, island area, sex-biased dispersal (tested only for *Erophylla*), or surface-level climatological winds. Gene flow among islands appears to be highly restricted, particularly for *M. waterhousii*, and we suggest that this species deserves increased taxonomic attention and conservation concern.

## Introduction

The factors promoting or restricting gene flow among populations have important consequences for a broad range of ecological and evolutionary processes [1], [2], [3]. Advances at the interface of GIS technology and population genetics have relaxed limitations associated with addressing the effects of large-scale, spatially dynamic variables and expanded our ability to rigorously analyze landscape features in the context of genetic structure [4], [5], [6]. As a result, the rapidly expanding field of landscape genetics [7], [8] incorporates data on landscape variables and knowledge of the study organism's dispersal ability, habitat preferences, movement patterns, and other pertinent ecological information into genetic studies.

A wide variety of demographic and physical processes can lead to asymmetric migration between populations [9], [10], [11]. Metapopulation theory predicts biased migration from highly productive ‘source’ populations into less productive ‘sink’ populations [12]. Differences in habitat area may also be related to biased migration between populations [13], assuming that larger areas have a greater amount of suitable habitat and, thus, larger population sizes. Distorted sex ratios, combined with sex-biased dispersal, can also lead to asymmetry in gene flow among populations [14]. In addition to these demographic forces, directional physical features (i.e. wind and water currents) can promote asymmetric gene flow in natural systems [15]. The consequences of these factors on gene flow are not straightforward, as they may interact with each other in complex ways [11], [14], [16]. The numerous implications of asymmetric gene flow include the ability of populations to adapt to local conditions, the evolution of species' ranges, metapopulation dynamics, biogeographical inference, and the design of effective conservation strategies [9], [10], [17], [18], [19].

To date, landscape genetics research has focused on systems where genetic connectivity is influenced by the spatial arrangement of different quality habitat and of physical barriers. In these systems, genetic connectivity among locations is predicted to be symmetric. In contrast, many natural systems are subject to strong directional forces that may have important effects on gene flow among populations. A valuable and logically appealing next step in landscape genetics is to extend the concept of ‘effective distance’ to situations with anisotropic forces. For example, wind and water currents have been demonstrated to influence gene flow of some passively dispersing organisms [20], [21], [22], [23], [24] (but see [25]) as well as oversea dispersal of terrestrial organisms [26]. The scarce research that has been conducted on the mechanistic basis of gene flow in systems with strong directional forces has focused on aquatic organisms [14], [20], [27]. Overall, empirical studies provide mixed conclusions on the overall occurrence of asymmetric gene flow in nature, even in systems with strong anisotropic forces.

Island systems, including those of the Caribbean basin, provide excellent opportunities to explore the mechanisms governing gene flow among populations. Despite little direct information about the ability of organisms to travel among islands, population genetic tools can provide insight into the patterns of colonization history and population dynamics. In particular, comparative studies provide a powerful means to reveal shared and disparate mechanisms mediating gene flow and to expose species differences that may have important conservation implications [28]. The primary goals of this study were to characterize patterns of genetic structure and gene flow in two lineages of bats in the Greater Antilles (*Erophylla sezekorni*/*bombifrons* and *Macrotus waterhousii*, Chiroptera: Phyllostomidae) and to explore the potential role of a variety of mechanisms that may drive these patterns. Specifically, we addressed the following questions: (1) What are the patterns of genetic structure throughout the Greater Antilles for these lineages of bats? (2) How much gene flow occurs among genetic populations in each of these taxa and is it symmetric? (3) Can instances of asymmetric gene flow be explained by differences in population size, island area, sex-biased dispersal, or climatological winds?

## Materials and Methods

### Sampling and data collection

#### Species

The two lineages of phyllostomid bats examined in this study are *Erophylla* and *Macrotus waterhousii*. The genus *Erophylla* contains two currently recognized species: *E. sezekorni* (the buffy flower bat), distributed in the western Greater Antilles (Cuba, Jamaica, the Caymans) and Bahamas, and *E. bombifrons* (the brown flower bat), which occurs in the eastern Greater Antilles (Hispaniola and Puerto Rico) [29]. Because of the low level of genetic differentiation between these lineages [30], [31], and because we were interested in the potential for maintained genetic connectivity between the species, for the purposes of this study, we pooled samples into a single lineage.

Bats of the genus *Macrotus* occur throughout the Bahamas and Greater Antilles (except Puerto Rico), as well as on the mainland from the southwestern United States south to Guatemala [32]. *Macrotus* is considered the basal genus of Phyllostomidae [33] and its origin has been dated at 28–34 million years [34], [35]. Of the two currently recognized species [29], *M. waterhousii* occurs in tropical dry forests of western Mexico and the Greater Antilles.

Adults of both genera weigh 12–20 g and have head/body lengths of 50–75 mm [32]. Both taxa roost typically in caves and colony sizes range from tens to thousands of individuals [36], [37] (T.H. Fleming & K.L. Murray, *unpublished data*). *Erophylla* bats are omnivores, feeding on nectar and fruits as well as insects [38] while species of *Macrotus* are considered to be insectivorous gleaners [39].

#### Sampling design and lab procedures

**Figure 1:**
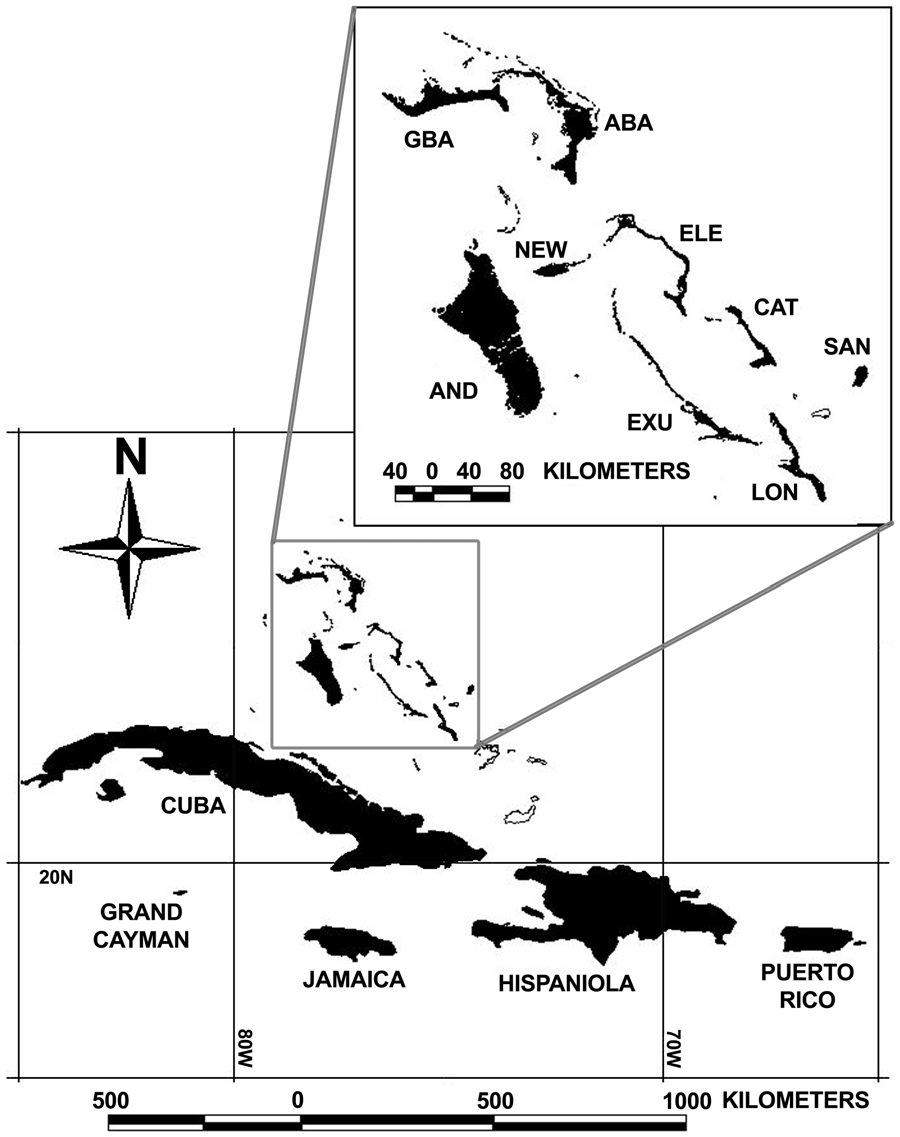
Map of the study area. Sampled islands are shown in black. Inset shows the Bahamas: ABA = Abaco, AND = Andros, CAT = Cat Island, ELE = Eleuthera, EXU = Exuma, GBA = Grand Bahama, LON = Long Island, NEW = New Providence and SAN = San Salvador. *Erophylla* was sampled on ABA, AND, CAT, Cuba, GBA, ELE, EXU, Hispaniola, Jamaica, LON, NEW, Puerto Rico, and SAN. *M. waterhousii* was sampled on ABA, CAT, Cuba, EXU, Grand Cayman, Hispaniola, Jamaica, and LON.

We obtained tissue samples from 13 islands for *Erophylla* (N = 293) and 8 islands for *M. waterhousii* (N = 190) throughout the Bahamas and Greater Antilles (Fig. 1). We captured bats with hand nets or mist nets, recorded their age, sex, reproductive status, body mass (g), forearm length (mm), and clipped a small piece of tissue (2–20 mg) from one wing membrane which was stored in 95% ethanol until lab analysis. Additional tissue samples were obtained from the American Museum of Natural History (Jamaica) or from the National Science Research Laboratory at the Museum of Texas Tech University (Jamaica and Cuba). Details of molecular markers, DNA extraction and other lab procedures are reported in [40].

### Data Analysis

#### Genetic diversity

We genotyped individuals of *Erophylla* at 12 microsatellite loci and *M. waterhousii* individuals at 10 microsatellite loci, and assessed genetic diversity using standard population genetic statistics including estimates of inbreeding coefficient (F_IS_) and pairwise F_ST_ values [41]. Because sex-biased dispersal can also contribute to asymmetric gene flow [14] and is sometimes extreme in bats [42], we tested *Erophylla* for evidence of sex-biased dispersal (sex data were insufficient to test *M. waterhousii*) through seven independent tests of differential genetic divergence between the sexes [43]. We used fstat version 2.9.3 [44] for all measures of genetic diversity, tests of sex-biased dispersal, pairwise population differentiation, conformation to Hardy-Weinberg proportions, and linkage equilibrium. We found no evidence for null alleles when data were screened using micro-checker [45].

#### Genetic Structure

We used two Bayesian clustering analyses to identify the number of genetically distinct populations (K) within the study area. In structure v.2 [46], we performed five independent trials of K = 1–13 (*Erophylla*) and K = 1–8 (*M. waterhousii*) for 20×10^5^ MCMC generations with a 20×10^4^ burn-in period. We selected the value of K (hereafter referred to as groups) to use in subsequent gene flow analyses based on the average maximum estimated log-likelihood of P(X|K) across trials. We assigned islands to each of the K groups based on the maximum estimated membership coefficient (*Q-*value) averaged for samples within islands. To provide an independent assessment of genetic structure, we used the group level analysis in baps v.5.1 [47], [48] to find the optimal number of genetic populations (K) for the two lineages. We inferred K as the smallest value after log-likelihood values reached a stable maximum [46]. To validate our pooling of *Erophylla* samples, we also ran these analyses separately for *E. sezekorni* and *E. bombifrons* (results not shown); results did not differ from the combined analyses.

#### GIS and Wind Data

**Figure 2:**
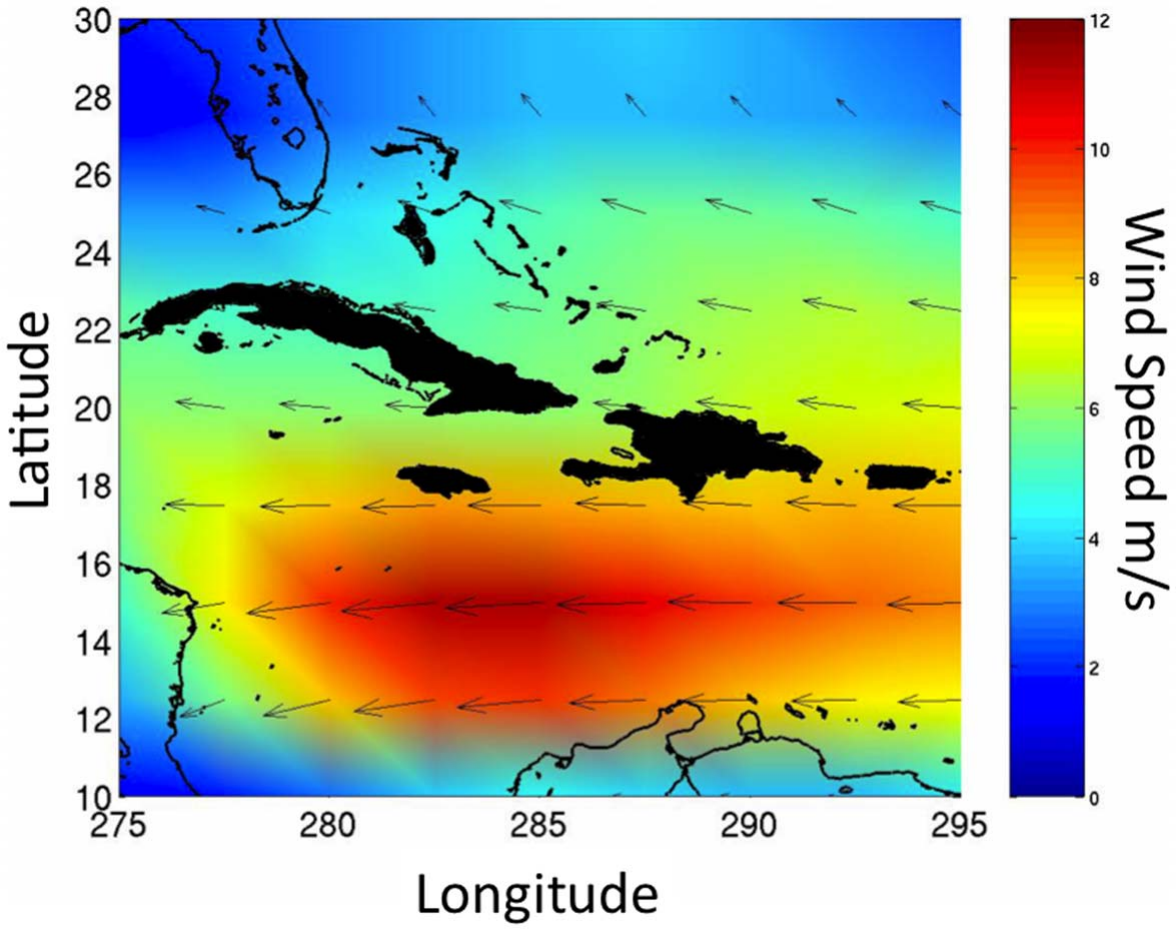
Wind surface of study area. A graphical representation of mean surface winds in the study area, as an example, during the month of June from 1948 to 2006. Right-hand scale shows wind magnitude in m/s.

**Figure 3:**
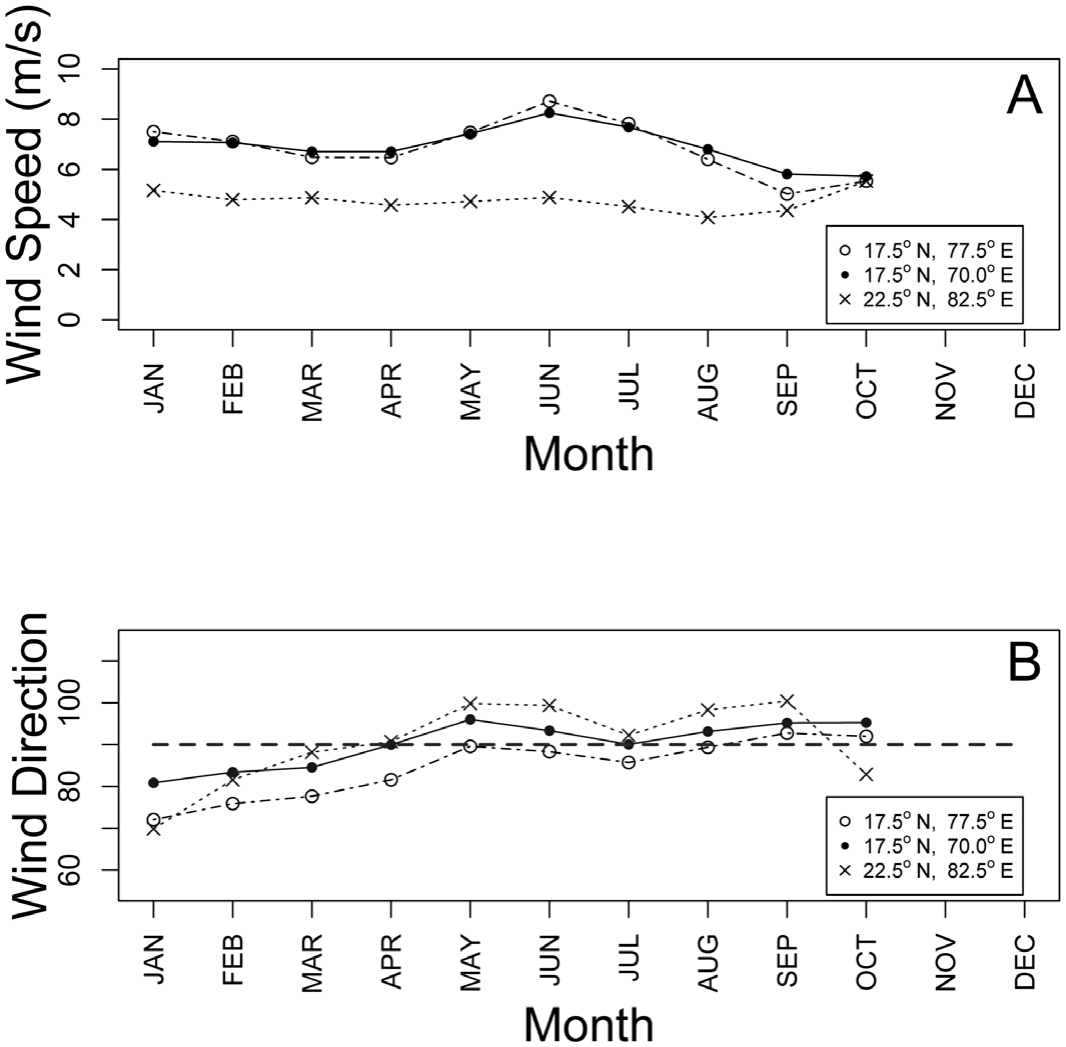
Seasonal wind patterns in the study area. Time series plot of mean wind magnitude (a) and mean wind direction (b) at three locations within the study area. Dashed line in (b) is included as a 90° (east) reference.

We compiled surface wind data from the National Climatic Data Center (ftp://ftp.cdc.noaa.gov/pub/Datasets), which includes monthly mean magnitude and direction from 1948 to 2005 at 2.5° lat/long (≈278 km^2^) resolution (Fig. 2). Data were not available for November and December. Climatological monthly mean winds were derived from the annual monthly means provided in the NCDC dataset by calculating the mean wind magnitude and direction for each month. To investigate seasonal variation in wind speed and direction, we generated time series plots of mean magnitude and direction at three locations within the study area (Fig. 3). Results presented here are from analyses performed using wind speed and direction averaged for all available months at each location. We assume that these wind data represent longer-term patterns that have remained relatively stable over evolutionary time. This assumption is based on the long-term stability of the particular meteorological dynamics driving the patterns [49].

#### Anisotropic Cost Analysis

First, we determined the Euclidean distance (D_GEO_) and azimuth of the shortest lines connecting all island pairs [50]. We then used anisotropic cost analysis to generate asymmetric and relative values of effective distance between features along these lines. Movement along the exact wind azimuth received a minimum cost to movement equal to the inverse of the wind speed. Deviations from the wind azimuth were treated by a standard anisotropic function [51], which incrementally penalized deviations from the wind azimuth with increased cost to movement. Effective distance, D_Wij_, was calculated as the product of the ‘friction’ due to wind and the Euclidean distance between islands. We evaluated the magnitude of asymmetry of effective distance using a normalized index of D_W_ asymmetry for all island pairs (*i*,*j*):
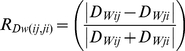
(1)R_Dw_ ranges from 0 (complete symmetry) to 1 (complete asymmetry).

#### Population size and gene flow

We estimated relative effective population size (θ) and levels of historical gene flow between genetic populations (M = *m*/μ) using maximum likelihood implemented in migrate v.2.1.3 [52]. Results from this program are best viewed as long-term estimates because it assumes mutation-migration-drift equilibrium, constant parameter values, and a per-locus mutation rate [53], [54]. We used the Brownian motion approximation to obtain initial parameter values. We developed a stepping stone model of gene flow by drawing the shortest possible line between each island pair. Island pairs were included in the stepping stone model if this line was not intersected by another island. Results presented for *Erophylla* are from 20 short chain searches (25×10^4^ trees sampled, 5×10^3^ trees recorded) followed by 3 long chain searches (25×10^5^ trees sampled, 5×10^4^ trees recorded) after a 10^4^ burn-in period. *M. waterhousii* results are from 15 short chain searches (2×10^4^ trees sampled, 10^3^ trees recorded) followed by 3 long chain searches (5×10^5^ trees sampled, 10^3^ trees recorded) after a 10^4^ burn-in period. The results of the final long chain searches were averaged over three independent runs. We identified asymmetric gene flow between genetic populations by examining 95% confidence intervals of M and calculated a normalized index of gene flow asymmetry (R_M_) for island or group pairs:
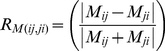
(2)R_M_ ranges from 0 (complete symmetry) to 1 (complete asymmetry) and pairs with overlapping 95% confidence intervals of migration rate were assigned R_M_ = 0. To determine if historic gene flow was biased from larger populations into smaller populations, we used linear regression to examine the relationship between θ and emigration. Similarly, we determined if historic gene flow was mediated by island size by examining the relationship between island area and migration rate.

We used bayesass v.1.3 [55] to obtain an estimate of the magnitude and direction of contemporary gene flow among populations. bayesass uses a MCMC algorithm to estimate the posterior probability distribution of the proportion of migrants from one population to another, M, without assuming genetic equilibrium. For each lineage, MCMC chains were run once for 10×10^6^ generations (2×10^6^ burn in) with a sampling frequency of 2×10^3^. To assess the reliability of estimated parameters, we ran four additional short MCMC runs for each lineage. Short runs were 5×10^6^ generations (1×10^6^ burn in) with the same sampling frequency. All other options were left at their default settings. In contrast to migrate, bayesass estimates all pairwise migration rates rather than a user-defined migration matrix.

Recognizing that populations may share alleles through either shared history or ongoing gene flow, we used coalescent analyses based on the non-equilibrium isolation-with-migration model to distinguish the influence of these two processes on population pairs. This model focuses on seven demographic parameters: the effective size of the ancestral population (*N*_A_), the effective sizes of the two daughter populations (*N*_1_ and *N*_2_), directional migration rates between the two daughter populations (*M*_1_ and *M*_2_), the divergence time for the two daughter populations (*t*), and the proportion of the ancestral population that founded daughter population 1 (*S*). All parameters, except for *S*, were estimated as mutation-scaled rates: *θ*_X_ = 4*N*_X_μ; τ = *t*μ; and *m*_X_ = *M*_X_/μ. We assumed a mutation rate of 10^−5^ mutations per locus per generation for these calculations. Populations were analyzed in pairwise combinations using the MCMC composite Bayesian/likelihood framework implemented in im v.3_5_2007 [56], [57]. For this application, our analyses focused on estimates of *S*, *t*, *M_1_* and *M_2_*. Uniform prior distributions were explored for *S* from 0–1, for *t* from 0–20, and for *m* from 0–20. These bounds were expanded when initial runs indicated that the full marginal posterior density was not being included. We emphasize that im produces marginal, not joint posteriors, so that failure of one parameter to converge is no reflection of uncertain inference for other parameters. Parameters were estimated from at least 3 jobs, each consisting of 10 Metropolis-Hastings coupled chains that were run for at least 5×10^5^ steps. Analyses were run on either a dispersed computing grid at the University of Arizona or at Cornell University's Computational Biology Service Unit (http://cbsuapps.tc.cornell.edu/index.aspx). Convergence on the underlying stationary distributions of the model parameters was assured by the use of multiple independent runs, many coupled chains, and long run times. Where convergence could not be obtained for specific parameters despite these efforts, we do not provide those parameter estimates.

#### Isolation by Distance

We tested our data for evidence of isolation by Euclidean distance (IBD_GEO_), between island groups. If mean surface winds mediate gene flow, we expected genetic differentiation to be more strongly correlated with a measure of effective distance than with Euclidean distance. We examined the correlation between genetic differentiation and the natural log of the minimum D_W_ for each pair of independent genetic populations. As an alternative approach, we used linear regression to evaluate the relationship between the index of migration asymmetry (R_M_) and the index of distance asymmetry (R_Dw_) for each group pair. Statistical significance of all IBD relationships was assessed using Mantel tests [58] performed for 10^4^ randomizations in fstat v.2.9.3 [44].

## Results

### Genetic Structure

#### Erophylla

**Figure 4:**
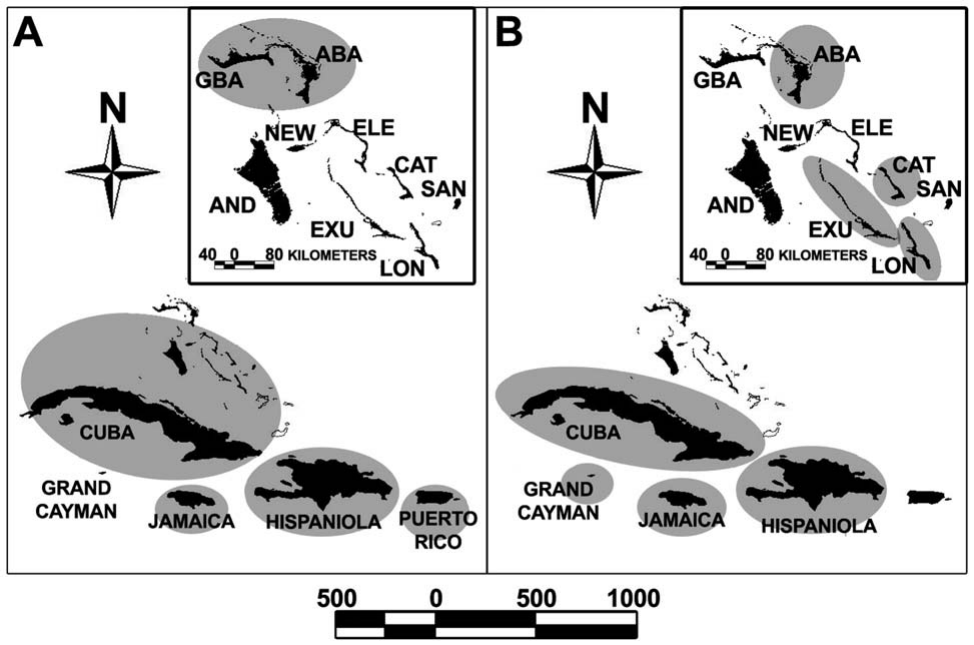
Genetic populations of two lineages of Greater Antillean bats. *Erophylla* (a) and *M. waterhousii* (b) as determined by structure analysis. Groups for *Erophylla* are: LBB = Little Bahama Bank, GBB = Great Bahama Bank, JAM = Jamaica, HIS = Hispaniola and PUE = Puerto Rico (see Fig. 1 for island abbreviations). Each island sampled for *M. waterhousii* represents an independent genetic population.

Estimated log likelihood values for 13 islands reached a maximum at K = 5 (mean −11436.7 +/− SD 1.99) (Text S1, C). Islands clustered into the following five groups: (1) Little Bahama Bank (LBB): Grand Bahama and Abaco, (2) Great Bahama Bank (GBB): Andros, Cat Island, Cuba, Eleuthera, Exuma, Long Island, New Providence and San Salvador, (3) Hispaniola (HIS), (4) Puerto Rico (PUE), and (5) Jamaica (JAM) (Fig. 4a). Because it contains Cuba and San Salvador, the group we denoted ‘GBB’ in this study is larger than the geologically defined Great Bahama Bank. The mean maximum proportion membership to genetic populations (island Q-values) was 0.60 (+/− SD 0.25). The baps analysis provided concordant results; log likelihood values reached a maximum for K = 5 (mean −11359.3 +/− SD 98.50) (Text S1, C) and the corresponding clusters were identical to those inferred from structure.

#### Macrotus

Estimated log likelihood values for 8 islands reached a maximum at K = 8 (mean −5681.82 +/− SD 1.21) in the structure analysis (Text S1, C). The mean maximum *Q*-values for islands was 0.84 (+/− SD 0.06), and all islands had a maximum average *Q*-value >0.76. Estimated log likelihood values from island samples in the baps analysis also reached a maximum for K = 8 (mean −5576.2 +/− SD 132.78) (Text S1, C). These results strongly suggest that all sampled islands belong to separate genetic populations (Fig. 4b).

### Genetic Diversity and Differentiation

Microsatellite marker characteristics for islands (*Macrotus*) and groups (*Erophylla*) are shown in Table 1 and pairwise F_st_ are provided with Euclidean distances between genetic populations in Table 2. In the *Erophylla* dataset, 142 total alleles were detected at 12 loci from all genotyped individuals (n = 293). Average observed heterozygosity over all loci was 0.66, and the estimated total F_IS_ over all loci was 0.039 (95% CI −0.007–0.072). Hardy-Weinberg proportions occurred in all groups (p>0.05), there was no evidence of linkage disequilibrium, and all pairwise tests of differentiation were significant (p<0.05) after Bonferroni correction. We found no statistical support for sex-biased gene flow (Table 3). In the *Macrotus* dataset, 108 total alleles were detected at 10 loci from the genotyped individuals (n = 190). Average observed heterozygosity over all loci was 0.67, Hardy-Weinberg proportions occurred at all sites, and we found no evidence of linkage disequilibrium. The estimated total F_IS_ over all loci was 0.034 (95% CI -0.009–0.059), and all pairwise tests of differentiation were significant (p<0.05) after Bonferroni correction. Allelic richness per locus was similar between the two lineages (*Macrotus* mean 6.5 +/− SD 1.49; *Erophylla* mean 6.9 +/− SD 1.84).

**Table 1:** Sample size (N), number of alleles (N_A_), number of private alleles (N_P_), mean allelic richness (A_R_), observed (H_o_) and expected (H_E_) heterozygosity and estimates of inbreeding coefficient (F_IS_) over all loci for *Erophylla* among groups and *M. waterhousii* among islands.

	N	N_A_	N_P_	A_R_	H_O_	H_E_	F_IS_
***Erophylla*** (groups)							
LBB	40	80	0	5.6	0.69	0.7	0.04
GBB	186	129	27	6.8	0.73	0.76	0.03
HIS	28	78	5	5.7	0.66	0.69	0.06
JAM	15	70	2	5.7	0.62	0.65	0.05
PUE	24	59	0	4.5	0.59	0.62	0.06
***M. waterhousii*** (islands)							
ABA	20	36	0	3.4	0.64	0.6	−0.07
CAT	26	54	0	4.7	0.69	0.72	0.04
CAY	11	38	2	3.8	0.69	0.72	0.06
CUB	11	52	1	5.1	0.73	0.77	0.06
EXU	39	61	1	5	0.69	0.72	0.04
HIS	32	73	9	5.6	0.73	0.77	0.05
JAM	24	70	17	6	0.71	0.78	0.09
LON	27	55	0	4.9	0.73	0.73	0.01

No significant (p<0.05) deviation from Hardy-Weinberg equilibrium were observed. See Figs. 1 and 4 for location abbreviations.

**Table 2:** F_ST_ (above diagonal) and Euclidean distance in km (below diagonal) for *Erophylla* among groups and for *M. waterhousii* among islands.

*Erophylla*					
	GBB	LBB	HIS	JAM	PUE
**GBB**	–	0.03	0.15	0.12	0.20
**LBB**	50	–	0.19	0.15	0.23
**HIS**	85	771	–	0.21	0.09
**JAM**	145	813	189	–	0.25
**PUE**	750	1315	114	946	–

*M. waterhousii*								
	ABA	CAT	CAY	CUB	EXU	HIS	JAM	LON
**ABA**	–	0.16	0.22	0.17	0.14	0.17	0.16	0.16
**CAT**	195	–	0.19	0.12	0.11	0.13	0.10	0.11
**CAY**	822	783	–	0.15	0.19	0.15	0.15	0.15
**CUB**	384	312	260	–	0.12	0.09	0.05	0.08
**EXU**	119	68	695	226	–	0.12	0.10	0.07
**HIS**	771	511	704	85	446	–	0.11	0.11
**JAM**	813	649	309	145	565	189	–	0.08
**LON**	305	49	740	210	24	365	536	–

See Fig. 1 for island and group abbreviations.

**Table 3:** Results for tests of sex-biased dispersal among groups in *Erophylla* (see Goudet *et al.* 2002 for more details about these tests).

	N	F_IS_	F_ST_	r	H_O_	H_E_	mA_I_	σ A_I_
*Females*	96	0.02	0.07	0.13	0.73	0.74	−0.49	11.15
Males	86	0.03	0.13	0.22	0.70	0.72	0.55	12.39
Total	182	0.02	0.10	0.18	0.71	0.73	–	–
p-value		ns	ns	ns	ns	ns	ns	ns

Statistical significance of differences between the sexes for these indices was accessed using the randomization procedure described by Goudet (2001) in FSTAT with 10^4^ randomizations.

### Gene Flow

**Figure 5:**
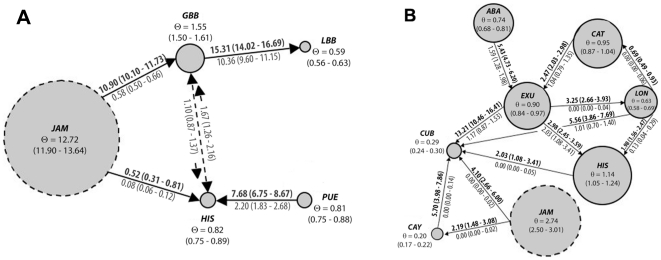
Estimated migration rates and estimated θ. Directional gene flow (plus 95% confidence intervals) and estimated θ between genetic populations of (a) *Erophylla* and (b) *M. waterhousii* based on estimates from migrate (see Fig. 1 for location abbreviations). Solid arrows represent significantly different (asymmetric) values, and bold values correspond to gene flow in the direction indicated. Dashed arrow represents symmetric gene flow for *Erophylla* but only instances of asymmetric gene flow are shown for *M. waterhousii* because of space limitations. Full results for both lineages can be found in Text S1, A. Circle sizes represent relative population size (θ) except for JAM, which reflects θ/2.

Historical migration rate (M = *m*/μ) and θ estimates between genetic populations of both lineages are shown in Fig. 5 and Text S1, A. Scaled migration rates obtained from migrate for *Erophylla* ranged from 0.08 to 15.31 (mean 5.04 +/− SD 5.23). Four of the five pairwise comparisons exhibited significantly asymmetric rates of gene flow based on non-overlapping 95% confidence intervals. The bayesass analysis suggested no detectable contemporary gene flow among genetic populations with one exception (Text S1, B). With both species of *Erophylla* pooled, we estimated M from PUE to HIS at 0.302 (95% CI 0.261–0.328). When the program was run using only data for *E. bombifrons*, however, gene flow from PUE to HIS was 0.019 (95% CI 0.00–0.07) and from HIS to PUE was 0.089 (95% CI 0.00–0.26). The percentage of changes accepted for these runs was 53.5 (+/− SD 0.055), falling within the range indicating acceptable algorithm performance [55].

The im analyses used a more parameter-rich, non-equilibrium model to simultaneously assess the effects of shared history and current gene flow on patterns of genetic similarity among islands (Table 4). Historical interactions such as the recent founding of one island population from another may be misinterpreted as recent gene flow under a model that assumes equilibrium conditions. The im model in particular allows inferences to be made about the direction of these population founding events through the population splitting parameter *S*. Assuming that such events involve only a small proportion of the ancestral population, we expect *S* to approach 1 when the common ancestor was located in the same place as daughter population 1, and to approach 0 when the ancestor was located in the same place as daughter 2 [59]. We used this basic assumption to infer the place of the most recent common ancestor (PMRCA). These same analyses also provided estimates of the time of these founding events as well as directional migration rates between the resulting daughter populations. In *Erophylla*, the GBB island cluster is indicated as a significant source founding other islands (LBB, Hispaniola, and Jamaica) approximately 30–40 thousand years ago (kya). An exception to this pattern is the founding of Hispaniolan *E. bombifrons* from the Puerto Rican population approximately 10 kya. Current levels of gene flow among islands was extremely low, averaging 11.24×10^−6^ (95% CI = 0.55–41.5×10^−6^), with only the GBB-LBB pair showing significantly asymmetric rates based on non-overlapping 95% confidence intervals.

**Table 4:** Results of im analyses for *Erophylla* and *M. waterhousii*, with 95% CI in parentheses.

Island pair (1–2)	PMRCA	Divergence time (kya)	M_1to2_ (×10^−6^)	M_2to1_ (×10^−6^)
***Erophylla***				
GBB-LBB	GBB	40 (34–52)	15.5 (10.3–24.0)	41.5 (33.8–85.6)
GBB-HIS	GBB	37 (33–50)	1.77 (1.10–5.83)	4.03 (1.23–7.57)
GBB-JAM	GBB	31 (25–34)	14.8 (10.7–21.9)	13.7 (8.37–27.8)
HIS-JAM	–	–	1.05 (0.10–5.45)	0.55 (0.10–8.60)
PUE-HIS	PUE	10 (8–14)	12.0 (8.99–148)	7.50 (2.60–114)
***M. waterhousii***				
ABA-EXU	EXU	15 (13–91)	3.83 (1.97–19.2)	12.2 (4.97–107)
EXU-CAT	EXU	19 (12–34)	7.23 (2.63–34.2)	0.77 (0.43–50.3)
EXU-LON	EXU	66 (46–367)	2.90 (1.20–35.3)	5.60 (1.70–39.8)
CAT-LON	–	30 (22–82)	0.83 (0.17–9.57)	11.0 (5.30–26.1)
CAT-HIS	CAT	273 (225–483)	1.20 (0.20–7.30)	2.70 (0.70–12.3)
LON-HIS	LON	279 (111–803)	3.70 (2.50–9.50)	0.50 (0.10–5.70)
LON-CUB	CUB	379 (269–1845)	8.70 (3.50–26.5)	0.10 (0.10–13.3)
EXU-CUB	EXU	119 (65–215)	0.55 (0.10–13.1)	7.30 (1.57–36.5)
EXU-HIS	–	–	2.03 (0.97–5.50)	0.90 (0.20–14.6)
JAM-HIS	JAM	247 (199–1771)	1.90 (0.30–4.30)	0.10 (0.10–2.30)
JAM-CUB	–	35 (22–238)	0.90 (0.30–37.1)	0.10 (0.10–10.1)
CAY-CUB	CAY	–	0.40 (0.25–21.1)	1.50 (0.30–52.4)
CAY-JAM	JAM	228 (130–926)	0.10 (0.10–12.8)	0.10 (0.10–16.3)
CAY-HIS	–	59 (19–389)	1.03 (0.37–22.6)	0.37 (0.10–15.8)
HIS-CUB	HIS	82 (63–700)	0.80 (0.10–7.45)	2.75 (1.10–11.9)

The place of the most recent common ancestor (PMRCA) was inferred from the population splitting parameter *S* (see text for details). Directional migration rates are given as fractions of migrating individuals per year. Missing estimates of PMRCA or divergence time could not be confidently estimated. See Fig. 4 for location abbreviations.

Migration rate scaled for mutation rate for *Macrotus* ranged from 0.00 to 13.20 (mean 1.98 +/− SD 2.74; Fig. 5). Twelve of the 15 island pairs included in the migration matrix displayed significantly asymmetric gene flow. Gene flow was relatively high and asymmetric between CUB and all other islands included in the migration matrix. Surprisingly, all of these instances showed immigration biased *toward* CUB (Fig. 5b). The relatively low sample size from Cuba (n = 11) may be limiting the number of observed alleles and therefore θ, which could result in a low number of estimated migrations (P. Beerli, *personal communication*). Two approaches were used to address this potential problem: (1) we randomly resampled 11 individuals from each other island and reran the analysis to investigate sample size bias, and (2) we reran the program including only samples from islands for which gene flow with Cuba was estimated (i.e. HIS, JAM and EXU) using the same settings as described above. These runs yielded qualitatively similar results in that migration was still biased toward CUB, suggesting adequate sample size to achieve consistent parameter estimates. The bayesass analysis estimated zero contemporary gene flow among all island pairs for *M. waterhousii* (Text S1, B). A low percentage of changes accepted for this analysis (<40%), however, hampers interpretation of these results. The im analyses of *M. waterhousii* (Table 4) were also consistent with extremely low levels of current gene flow (mean M = 2.74×10^−6^; 95% CI = 0.10–12.2×10^−6^). While we found no statistical support for asymmetric gene flow between any pair of islands with the im analysis, the 95% confidence intervals were quite large in most cases, reducing the power of this assessment. Instead, these analyses attributed most genetic similarity between islands to common ancestry and historical founding events. In general, the oldest of these founding events (273–379 kya; 95% CI = 111–1845 kya) connect islands of the Bahamas with near islands of the Greater Antilles (Hispaniola and Cuba). Except for Jamaica, founding events between islands of the Greater Antilles appear to be significantly more recent (59–82 kya; 95% CI = 19–700 kya). Founding events among islands of the Bahamas are, in most cases, even more recent (15–66 kya; 95% CI = 12–367 kya) with Exuma indicated as a significant source population.

Theta values estimated by migrate were not correlated with historical emigration rate for either lineage (*Erophylla*: R^2^ = 0.004, p>0.05; *Macrotus*: R^2^ = 0.005, p>0.05). Additionally, neither θ nor M were correlated with the island (*M. waterhousii*) or total group area (*Erophylla*) (p>0.05 in all cases). These results suggest that asymmetric gene flow is not mediated by differences in estimated population size or island area.

### Isolation by distance

**Figure 6:**
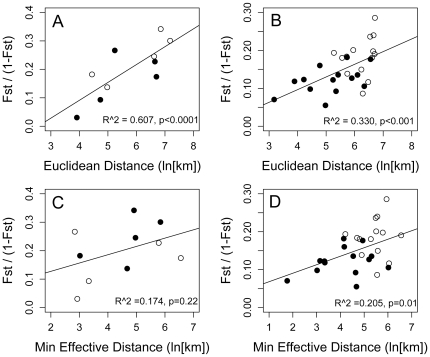
Isolation by distance. Isolation by distance plots using Euclidean (a,b) and *effective* (c,d) distance among genetic populations of *Erophylla* (a,c) and *M. waterhousii* (b,d). Filled circles represent pairwise comparisons included in the migrate migration matrix and open circles represent pairwise comparisons excluded from this analysis. Regression lines are based on all points shown.

Isolation by distance plots among genetic populations for each lineage using both Euclidean and *effective* distance metrics are shown in Fig. 6. We detected significant IBD_GEO_ for *Erophylla* when considering all islands separately (data not shown, R^2^ = 0.537, p<0.0001) as well as among groups (R^2^ = 0.607, p<0.001), and for *M. waterhousii* among islands (R^2^ = 0.330, p<0.001). Minimum pairwise effective distance between groups was not significantly correlated with genetic differentiation for *Erophylla* (R^2^ = 0.174, p = 0.22) but was for *M. waterhousii* (R^2^ = 0.205, p = 0.01). However, genetic differentiation was more strongly correlated with Euclidean distance than effective distance in both cases. There were no significant relationships between the index of distance asymmetry (R_Dw_) and the index of migration asymmetry (R_M_) for either *Erophylla* or *M. waterhousii* (p>0.05 for all cases). From these results, we conclude that both lineages displayed significant IBD_GEO_ at the scale of this study but our measure of effective distance did not provide an explanation of the distribution of genetic diversity or the instances of asymmetric gene flow.

## Discussion

The results of this study reveal substantially different patterns of genetic structure for *Erophylla* and *M. waterhousii* in the Greater Antilles and Bahamas. While the estimated magnitude of historic gene flow between genetic populations was generally greater for *Erophylla* than for *M. waterhousii*, contemporary gene flow appears to be highly restricted among populations in both lineages. Additionally, parts of our analyses suggested an equally high incidence of asymmetric historic gene flow in both lineages. While this finding could have profound consequences for evolution and conservation of species, we found no evidence that a directional bias of historical gene flow was related to effective population size, island area, sex-biased dispersal, or surface-level trade winds. Results from the IM analysis suggested that these patterns might potentially be a result of shared ancestry rather than gene flow among island populations. Overall, our results support Hedges' [60] view that the effect of distance is the most important determinant of dispersal for actively dispersing organisms in the Caribbean.

### Genetic Structure

*Erophylla* exhibited less genetic structure than we had anticipated given its island endemism. Poor dispersal ability may lead to endemism through an inability to colonize distant sites, but this does not appear to be the case for *Erophylla*. A similarly counterintuitive pattern of genetic structure of an island endemic bat was found by Roberts [61], demonstrating that the vagaries of environmental history can be important determinants of genetic structure and may not always result in patterns consistent with expectations of limited dispersal ability. As expected, the most striking genetic differentiation we detected for *Erophylla* was between bats from the eastern islands of the range of the genus (Hispaniola and Puerto Rico) versus the rest of the Greater Antilles and Bahamas. Fleming *et al.* [31] used D-loop mtDNA sequence data to demonstrate a corresponding subdivision within *Erophylla*. Haplotypes were shared extensively within the two clades, and two out of 34 total haplotypes (6%) were shared between clades. Based on these data, the authors rejected the hypothesis of island monophyly, suggesting that gene flow may still be occurring between the two clades. In the current study, we found no evidence of current gene flow between these two clades, consistent with the current taxonomy of the genus [29]. However, this genetic division and the lack of current gene flow remains perplexing given the relatively short distance between Hispaniola and Cuba (approximately 85 km). Within the eastern and western clades, however, genetic differentiation is relatively low, suggesting relatively recent isolation (e.g., 10 kya between Puerto Rico and Hispaniola). This observation could reflect greater island connectivity during periods of lower sea levels in the Pleistocene. Despite our analyses suggesting limited current gene flow among genetic populations, additional evidence for connectivity comes from a lack of recorded local extinctions of *Erophylla*. Throughout its range, no extirpated populations have been recorded on the islands throughout the Greater Antilles and Bahamas where *Erophylla* occurs in the fossil record [62]. More information on habitat use would be helpful to assess the extinction risk in this lineage [63], [64].

Unlike the results for *Erophylla* but concordant with the results of Fleming *et al.* [31], island populations of *M. waterhousii* appear to be genetically isolated from one another. The strong genetic subdivisions between island populations may be indicative of this species' sedentary lifestyle and long residence in the Greater Antilles. In contrast to *Erophylla*, Morgan [62] reported that *M. waterhousii* has become extinct on six of the 30 islands from which it is included in the fossil record. These findings signify a geographical range contraction since the late Quaternary and suggest that recolonization events between islands are rare in this lineage.

### Gene Flow

Despite differences in the overall amount of genetic differentiation among populations between these two lineages, results from migrate suggest asymmetric historical gene flow among a majority of pairwise comparisons (∼80% in both lineages). Meanwhile, the results of the bayesass analysis suggest that contemporary gene flow is highly restricted among genetic populations for both lineages. It is possible that the high degree of historic gene flow asymmetry inferred from the migrate results is actually an artifact generated from effects of shared ancestry. The results of our im analyses do not generally support an inference of high levels of asymmetric gene flow. There are likely two effects leading to these seemingly discordant results. First, because migrate uses an equilibrium model, effects of shared genetic history among islands, specifically the recent founding of one island population from another, may emerge as asymmetric migration rates in parameter estimation. Second, some actual instances of asymmetry may not be detected in im using the criterion of non-overlapping 95% confidence intervals because the estimates of these values from im are quite large. Recognizing these possibilities, we focus our discussion below on possible explanations for the high levels of asymmetric historical gene flow observed in the migrate analysis.

Previous studies on a variety of taxa provide mixed conclusions on the mechanisms leading to asymmetric gene flow [11], [14], [16], [20]. Invariably, interpreting patterns of genetic structure involves a variety of factors that can interact in unpredictable ways. In this study, we focused on three possible factors that could lead to asymmetric gene flow among populations: (1) unequal population sizes, (2) sex-biased dispersal and (3) surface-level trade winds.

#### Demographic Factors

Sex-biased dispersal is unlikely to be a contributing factor to asymmetric gene flow in this system. The sex ratio of *Erophylla* appears to 1∶1 [30] and none of the seven tests for sex-biased dispersal indicated a disparity in the gene flow contributed by the two sexes.

Estimated relative effective population size (θ) was not correlated with the index of emigration for either species, suggesting that larger populations do not always act as sources in this system. Additionally, island area was not correlated with relative effective population size (θ) or the directional bias of historic gene flow. In fact, despite its ranking as third largest island area, estimates of θ for Jamaica were considerably higher than the group or island with the second largest θ (by a factor of 8 for *Erophylla* and 2 for *M. waterhousii*) (Fig. 5). Sampling bias might explain these results if sample size had been greater on Jamaica than other islands [52], but this was not the case. In fact, samples from Jamaica were collected from bats inhabiting a single cave. One implication of this pattern is that Jamaica was the site of origin of both of these lineages (or, in the case of *M. waterhousii*, the port of entry into the Caribbean from Mexico). The results from this study suggest straightforward dispersal of *Erophylla* from Jamaica to GBB (presumably first to Cuba) and then to Hispaniola. Our observation of decreasing θ values in *Erophylla* away from Jamaica along the dispersal pathway also fit this model. The pattern for *M. waterhousii* is not as clear, but one interpretation of differences in θ's among islands is that Jamaica was colonized first, followed by Hispaniola and then Cuba and the Bahamas [65].

#### The Effect of Wind

We expected the strongly asymmetric force of wind present in this system to play a role in mediating gene flow among populations. We hypothesized that surface level trade winds may lead to asymmetric gene flow because of their effects on the flight dynamics of volant organisms [66], [67], [68]. The results of this study, however, do not provide evidence that gene flow is mediated by wind for either of these lineages. In spite of these results, we cannot entirely rule out a potential mechanistic role of wind. We designed our approach to examine if instances of asymmetric gene flow could be attributed to winds hypothetically encountered by a dispersing bat *on average*. Perhaps the spatial and temporal resolution of the wind data used in this study does not adequately capture the overall effect on the movement of bats between islands. However, because the results from the migrate analysis are best viewed as long-term parameter estimates [69], it seems appropriate to examine them in the context of long-term averages of wind. It is possible that that regional wind patterns over evolutionary time scales differed somewhat from those used in this study (i.e., the past 60 years). This is unlikely, however, given that the general wind patterns observed in this study (NE trade winds) result from stable meteorological dynamics [49].

Wind may not mediate gene flow in these lineages if their flight speeds are greater than the wind speeds they encounter during movements between islands. To investigate seasonal variation in wind speed and direction, we generated time series plots of mean magnitude and direction at three locations within the study area (Fig. 3). Inferences of bat flight performance and habitat use can be made from wing morphology [70], [71]. Jennings *et al.* [72] described the wing morphology of *Erophylla* as suited for maneuverable but not fast or efficient flight. Similarly, Valdivieso *et al.* [73] inferred that *Erophylla* is suited for short bursts of flight based on their study of lactate dehydrogenase isozymes. *M. waterhousii* has similar wing morphology as *Erophylla* [70]. Unfortunately, flight speed data do not currently exist for either *Erophylla* or *M. waterhousii*. For comparative purposes, *Leptonycteris curasoae*, a strong flying phyllostomid, commutes between day roost and feeding areas at an average air speed of 8.2 m/s [74]. This is slightly greater than the mean wind speeds recorded in the study area (Fig. 3). If flight speeds of *E. sezekorni* and *M. waterhousii* are comparable to those of *L. curasoae*, then perhaps the mean wind does not represent a large enough additional energetic cost to affect their movement patterns. However, these bats are likely to be slower flyers than *L. curasoae*, an exceptionally fast flyer (T. H. Fleming, *personal observation*). Seasonal variation in wind speed may be important if periods of peak wind speeds correspond to bat migration or dispersal. Repeating our analyses using data from June (the month of peak wind speeds) provided comparable results in that Euclidean distance accounted for genetic differentiation better than effective distance.

The results of this study do not support our hypothesis that gene flow is mediated by surface-level trade wind for either of these lineages. Wind may still play a role, however, if idiosyncratic events (i.e., hurricanes) contribute disproportionately to stochastic, long-distance dispersal of bats. Hurricanes have historically been a major climatological presence in the study area with an average of three hurricane-strength events per year over the past 500 years [75]. While it is feasible that these storms affect long distance dispersal of bats and other organisms in the region [31], the exact mechanism is difficult to resolve. Hurricanes can lead to a substantial decline in population sizes due to a combination of direct and indirect effects (i.e. decimated food supplies or destroyed roosting structures) [76], [77]. There is some anecdotal evidence of long distance dispersal of bats, including *Erophylla*, following hurricanes [78] (T.H. Fleming, *personal observation*), but we do not know the exact mechanism leading to these ‘transplants’. Interestingly, the annual peak in hurricane activity in the study area occurs in late summer, which coincides with the time when juvenile bats become volant and are potentially dispersing from their natal colonies (T. H. Fleming, *personal observation*).

### Comparison of Lineages

What differences are responsible for producing the two lineage's different patterns of genetic structure and gene flow observed in this study? Divergent patterns of genetic structure have been reported among other phyllostomid species in the Lesser Antilles (e.g. *Ardops nichollsi*, *Brachyphylla cavernarum*, and *Artibeus jamaicensis*) [28]. In that study, the observed genetic patterns were attributed to differential rates of gene flow among islands, incomplete lineage sorting, and ecological differences between these taxa. The particular mechanism driving differential rates of gene flow among islands, however, remains uncertain. Similarly, Zink [79] found little evidence for phylogeographic congruence among North American birds, even for ecologically similar taxa. Heaney [80] recently made a general argument that closely related species in a single region often have very different patterns of gene flow at least partially due to varied ecological responses to environmental history. Consistent with this prediction, Roberts [61] found contrasting patterns of genetic structure among three species of pteropodid bats in the Philippines. While somewhat surprising given these species' distributions, the genetic patterns appeared to be related to differences in habitat preference that can be linked to environmental history.

The strong genetic differentiation among island populations of *M. waterhousii*, implies limited over-water dispersal ability in this species. Two studies on the biological correlates of extinction risk in bats [63], [64] suggest that breadth of habitat use and wing morphology are the best predictors of extinction risk in bats. While wing morphology is similar between the two lineages of bats examined in this study, more information is required to determine if differences in habitat use can account for the different extinction patterns of these two lineages. Overall, our results suggest that island populations, particularly of *M. waterhousii*, deserve greater taxonomic attention and conservation concern.

### Conclusion

The results of this study reveal substantially different patterns of genetic structure for *Erophylla* and *M. waterhousii* in the Greater Antilles and Bahamas. While the estimated magnitude of historic gene flow between genetic populations was generally greater for *Erophylla* than for *M. waterhousii*, contemporary gene flow appears to be highly restricted among populations in both lineages. Additionally, some of our analyses suggested an equally high incidence of asymmetric historic gene flow in both lineages. We found no evidence, however, that directional bias of historical gene flow was related to effective population size, island area, sex-biased dispersal, or surface-level trade winds. Results from the im analysis suggested these patterns are potentially a result of shared ancestry rather than gene flow among island populations. Overall, our results support Hedges' [60] view that the effect of distance is the most important determinant of dispersal for actively dispersing organisms in the Caribbean.

## Supporting Information

Text S1 Includes full results from the migrate and bayesass analyses, as well as negative log likelihood for the structure and baps analyses.
(DOC)
