# Supplementary material for: Exploring Demographic, Physical, and Historical Explanations for the Genetic Structure of Two Lineages of Greater Antillean Bats
Source: PLoS One. 2011 Mar 21;6(3):e17704. doi: 10.1371/journal.pone.0017704 (PMC3061861; doi:10.1371/journal.pone.0017704)
Supplement: Text S1 — Includes full results from the migrate and bayesass analyses, as well as negative log likelihood for the structure and baps analyses. (DOC) [file pone.0017704.s001.doc]

**SUPPORTING INFORMATION S1.**

**Supporting Information S1,** **A.** Theta (N_e_µ) (diagonal, in bold) and M (*m*/µ) (off diagonal) among genetic populations of (a) *Erophylla* and (b) *M. waterhousii* as determined by migrate; 95% confidence intervals are given in parentheses. Migration rates represent gene flow *FROM* the column locations *TO* the row locations. Group abbreviations for *Erophylla* are: LBB=Little Bahama Bank, GBB=Great Bahama Bank, JAM=Jamaica, HIS=Hispaniola and PUE=Puerto Rico. Island abbreviations for *M. waterhousii* are: ABA=Abaco, CAT=Cat Island, CAY=Grand Cayman, CUB=Cuba, EXU=Exuma, HIS=Hispaniola, JAM=Jamaica, and LON=Long Island.

a.

|  | **LBB** | **GBB** | **HIS** | **JAM** | **PUE** |
| --- | --- | --- | --- | --- | --- |
| LBB | **0.59**  **(0.56-0.63)** | 15.31  (14.02-16.69) | -- | -- | -- |
| **GBB** | 10.36  (9.60-11.15) | **1.55**  **(1.50-1.61)** | 1.10  (0.87-1.37) | 10.90  (10.10-11.73) | -- |
| **HIS** | -- | 1.67  (1.26-2.16) | **0.82**  **(0.75-0.89)** | 0.52  (0.31-0.81) | 7.68  (6.75-8.67) |
| **JAM** | -- | 0.58  (0.50-0.66) | 0.08  (0.06-0.12) | **12.72**  **(11.90-13.64)** | -- |
| **PUE** | -- | -- | 2.20  (1.83-2.68) | -- | **0.81**  **(0.75-0.88)** |

b.

|  | ABA | CAT | CAY | CUB | EXU | HIS | JAM | LON |
| --- | --- | --- | --- | --- | --- | --- | --- | --- |
| ABA | **0.74**  **(0.68-0.81)** | -- | -- | -- | 1.59  (1.28-1.98) | -- | -- | -- |
| **CAT** | -- | **0.95**  **(0.87-1.04)** | -- | -- | 1.04  (0.79-1.35) | 0.00  (0.00-0.03) | -- | 0.69  (0.49-0.93) |
| **CAY** | -- | -- | **0.20**  **(0.17-0.22)** | 0.00  (0.00-0.14) | -- | 1.80  (1.18-2.59) | 2.19  (1.48-3.08) | -- |
| **CUB** | -- | -- | 5.70  (3.98-7.86) | **0.29**  **(0.24-0.30)** | 13.21  (10.46-16.41) | 2.03  (1.08-3.41) | 4.10  (2.66-6.00) | 5.56  (3.86-7.69) |
| **EXU** | 5.43  (4.73-6.20) | 2.47  (2.03-2.98) | -- | 1.17  (0.87-1.53) | **0.90**  **(0.84-0.97)** | 0.35  (0.20-0.56) | -- | 0.00  (0.00-0.04) |
| **HIS** | -- | 0.00  (0.00-0.05) | 0.95  (0.67-1.30) | 0.00  (0.00-0.05) | 2.98  (2.45-3.59) | **1.14**  **(1.05-1.24)** | 0.84  (0.58-1.18) | 1.98  (1.56-2.47) |
| **JAM** | -- | -- | 0.00  (0.00-0.02) | 0.00  (0.00-0.02) | -- | 0.79  (0.64-0.96) | **2.74**  **(2.50-3.01)** | -- |
| **LON** | -- | 0.00  (0.00-0.06) | -- | 1.01  (0.70-1.40) | 3.25  (2.66-3.93) | 0.13  (0.04-0.29) | -- | **0.63**  **(0.58-0.69)** |

**Supporting Information S1, B**. Estimated current gene flow, M, between genetic populations of *Erophylla* (a) and *M. waterhousii* (b) as estimated by bayesass. Values that differ significantly from what would be expected if no information were contained within the data are shown in bold.

a.

| *From* | *To* | M +/- SD | 95% CI |
| --- | --- | --- | --- |
| **GBB** | **GBB** | **0.992 +/- 0.007** | **0.981 - 1** |
| JAM | GBB | 0.002 +/- 0.002 | 0 - 0.007 |
| HIS | GBB | 0.001 +/- 0.001 | 0 - 0.004 |
| PUE | GBB | 0.001 +/- 0.001 | 0 - 0.004 |
| LBB | GBB | 0.004 +/- 0.006 | 0 - 0.014 |
| GBB | JAM | 0.022 +/- 0.022 | 0 - 0.082 |
| **JAM** | **JAM** | **0.953 +/- 0.029** | **0.88 - 0.993** |
| HIS | JAM | 0.007 +/- 0.01 | 0 - 0.035 |
| PUE | JAM | 0.007 +/- 0.01 | 0 - 0.036 |
| LBB | JAM | 0.01 +/- 0.014 | 0 - 0.053 |
| GBB | HIS | 0.008 +/- 0.01 | 0 - 0.03 |
| JAM | HIS | 0.006 +/- 0.008 | 0 - 0.029 |
| **HIS** | **HIS** | **0.678 +/- 0.011** | **0.667 - 0.706** |
| **PUE** | **HIS** | **0.302 +/- 0.018** | **0.261 - 0.328** |
| LBB | HIS | 0.006 +/- 0.008 | 0 - 0.031 |
| GBB | PUE | 0.003 +/- 0.006 | 0 - 0.018 |
| JAM | PUE | 0.003 +/- 0.006 | 0 - 0.02 |
| HIS | PUE | 0.003 +/- 0.006 | 0 - 0.02 |
| PUE | **PUE** | **0.987 +/- 0.012** | **0.953 - 1** |
| LBB | PUE | 0.003 +/- 0.006 | 0 - 0.019 |
| GBB | LBB | 0.003 +/- 0.006 | 0 - 0.02 |
| JAM | LBB | 0.002 +/- 0.003 | 0 - 0.01 |
| HIS | LBB | 0.002 +/- 0.003 | 0 - 0.011 |
| PUE | LBB | 0.002 +/- 0.003 | 0 - 0.009 |
| **LBB** | **LBB** | **0.992 +/- 0.008** | **0.972 - 1** |

b.

| *From* | *To* | M +/- SD | 95% CI |
| --- | --- | --- | --- |
| **EXU** | **EXU** | **0.987 +/- 0.012** | **0.962 - 1** |
| ABA | EXU | 0.002 +/- 0.004 | 0 - 0.014 |
| CAT | EXU | 0.003 +/- 0.006 | 0 - 0.013 |
| LON | EXU | 0.002 +/- 0.004 | 0 - 0.013 |
| CUB | EXU | 0.002 +/- 0.003 | 0 - 0.012 |
| JAM | EXU | 0.002 +/- 0.004 | 0 - 0.012 |
| HIS | EXU | 0.002 +/- 0.003 | 0 - 0.011 |
| CAY | EXU | 0.002 +/- 0.003 | 0 - 0.01 |
| EXU | ABA | 0.003 +/- 0.006 | 0 - 0.022 |
| **ABA** | **ABA** | **0.983 +/- 0.016** | **0.943 - 0.999** |
| CAT | ABA | 0.002 +/- 0.006 | 0 - 0.02 |
| LON | ABA | 0.002 +/- 0.006 | 0 - 0.021 |
| CUB | ABA | 0.002 +/- 0.005 | 0 - 0.019 |
| JAM | ABA | 0.002 +/- 0.005 | 0 - 0.017 |
| HIS | ABA | 0.002 +/- 0.006 | 0 - 0.018 |
| CAY | ABA | 0.002 +/- 0.005 | 0 - 0.018 |
| EXU | CAT | 0.012 +/- 0.02 | 0 - 0.052 |
| ABA | CAT | 0.005 +/- 0.01 | 0 - 0.035 |
| **CAT** | **CAT** | **0.966 +/- 0.031** | **0.9 - 1** |
| LON | CAT | 0.005 +/- 0.009 | 0 - 0.033 |
| CUB | CAT | 0.003 +/- 0.006 | 0 - 0.023 |
| JAM | CAT | 0.003 +/- 0.006 | 0 - 0.02 |
| HIS | CAT | 0.003 +/- 0.006 | 0 - 0.021 |
| CAY | CAT | 0.003 +/- 0.006 | 0 - 0.019 |
| EXU | LON | 0.008 +/- 0.013 | 0 - 0.039 |
| ABA | LON | 0.005 +/- 0.009 | 0 - 0.024 |

| *From* | *To* | M +/- SD | 95% CI |
| --- | --- | --- | --- |
| CAT | LON | 0.004 +/- 0.008 | 0 - 0.034 |
| **LON** | **LON** | **0.972 +/- 0.023** | **0.919 - 0.999** |
| CUB | LON | 0.003 +/- 0.005 | 0 - 0.023 |
| JAM | LON | 0.003 +/- 0.005 | 0 - 0.02 |
| HIS | LON | 0.003 +/- 0.006 | 0 - 0.017 |
| CAY | LON | 0.003 +/- 0.006 | 0 - 0.018 |
| EXU | CUB | 0.018 +/- 0.025 | 0 - 0.088 |
| ABA | CUB | 0.025 +/- 0.033 | 0 - 0.159 |
| CAT | CUB | 0.013 +/- 0.019 | 0 - 0.079 |
| LON | CUB | 0.127 +/- 0.083 | 0 - 0.282 |
| **CUB** | **CUB** | **0.731 +/- 0.076** | **0.668 - 0.804** |
| JAM | CUB | 0.045 +/- 0.056 | 0 - 0.167 |
| HIS | CUB | 0.02 +/- 0.026 | 0 - 0.081 |
| CAY | CUB | 0.02 +/- 0.027 | 0 - 0.104 |
| EXU | JAM | 0.004 +/- 0.008 | 0 - 0.029 |
| ABA | JAM | 0.002 +/- 0.005 | 0 - 0.02 |
| CAT | JAM | 0.003 +/- 0.006 | 0 - 0.017 |
| LON | JAM | 0.004 +/- 0.008 | 0 - 0.029 |
| CUB | JAM | 0.003 +/- 0.006 | 0 - 0.019 |
| **JAM** | **JAM** | **0.979 +/- 0.02** | **0.929 - 1** |
| HIS | JAM | 0.002 +/- 0.005 | 0 - 0.018 |
| CAY | JAM | 0.003 +/- 0.007 | 0 - 0.02 |
| EXU | HIS | 0.002 +/- 0.005 | 0 - 0.019 |
| ABA | HIS | 0.002 +/- 0.005 | 0 - 0.019 |
| CAT | HIS | 0.002 +/- 0.005 | 0 - 0.016 |
| LON | HIS | 0.003 +/- 0.006 | 0 - 0.02 |

| *From* | *To* | M +/- SD | 95% CI |
| --- | --- | --- | --- |
| CUB | HIS | 0.002 +/- 0.004 | 0 - 0.017 |
| JAM | HIS | 0.002 +/- 0.004 | 0 - 0.017 |
| **HIS** | **HIS** | **0.985 +/- 0.015** | **0.946 - 0.999** |
| CAY | HIS | 0.003 +/- 0.006 | 0 - 0.02 |
| EXU | CAY | 0.004 +/- 0.009 | 0 - 0.033 |
| ABA | CAY | 0.004 +/- 0.009 | 0 - 0.032 |
| CAT | CAY | 0.004 +/- 0.009 | 0 - 0.029 |
| LON | CAY | 0.004 +/- 0.009 | 0 - 0.031 |
| CUB | CAY | 0.004 +/- 0.009 | 0 - 0.031 |
| JAM | CAY | 0.004 +/- 0.009 | 0 - 0.03 |
| HIS | CAY | 0.004 +/- 0.009 | 0 - 0.03 |
| **CAY** | **CAY** | **0.974 +/- 0.024** | **0.91 - 0.999** |


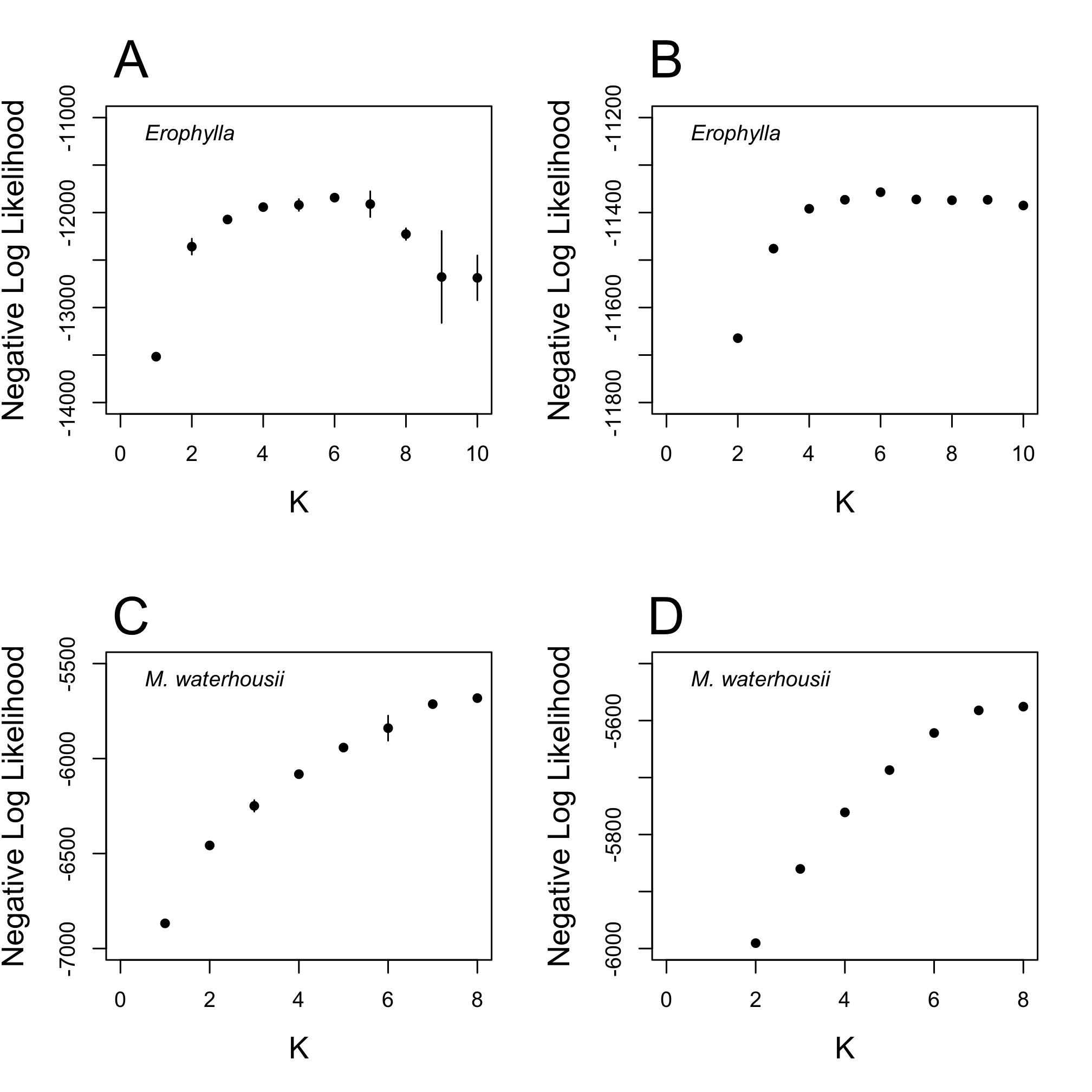


**Supporting Information S1, C.** Negative log likelihood values plotted for various values of K provided by structure (A,C) and baps (B,D) for *Erophylla* and *M. waterhousii*. Error bars represent standard deviation and scale varies between panels.
